# Concomitant mitral regurgitation in patients with low-gradient aortic stenosis: an analysis from the German Aortic Valve Registry

**DOI:** 10.1007/s00392-022-02067-2

**Published:** 2022-08-19

**Authors:** Brunilda Alushi, Stephan Ensminger, Eva Herrmann, Ümniye Balaban, Timm Bauer, Andreas Beckmann, Sabine Bleiziffer, Helge Möllmann, Thomas Walther, Raffi Bekeredjian, Christian Hamm, Friedhelm Beyersdorf, Stephan Baldus, Andreas Boening, Volkmar Falk, Holger Thiele, Christian Frerker, Alexander Lauten

**Affiliations:** 1 Department of Cardiovascular Diseases, Campus Benjamin Franklin, Charité-Universitätsmedizin Berlin, Corporate Member of Freie Universität Berlin and Humboldt-Universität zu Berlin and German Centre for Cardiovascular Research (DZHK) Berlin Site, Hindenburgdamm 30, 12200 Berlin, Germany; 2 Department of Internal Medicine and Cardiology, Zollernalbklinik, Balingen, Germany; 3 Department of Cardiac and Thoracic Vascular Surgery, University Heart Center Lübeck, Lübeck, Germany; 4 Institute of Biostatistics and Mathematical Modelling at Goethe University, Frankfurt am Main, Germany; 5 German Center for Cardiovascular Research, (DZHK), Partner Site Rhine Main, Frankfurt am Main, Germany; 6 Department of Cardiology, Sana Klinikum Offenbach, Offenbach, Germany; 7 German Society for Thoracic and Cardiovascular Surgery, Langenbeck-Virchow-Haus, Berlin, Germany; 8 Clinic for Thoracic and Cardiovascular Surgery, Herz- und Diabeteszentrum NRW, Ruhr Universität Bochum, Bad Oeynhausen, Germany; 9 Medizinische Klinik I, St.-Johannes-Hospital Dortmund, Dortmund, Germany; 10 Department of Cardiac Surgery, Goethe University Hospital, Frankfurt am Main, Germany; 11 Department of Cardiology, Robert-Bosch-Hospital, Stuttgart, Germany; 12 Department of Cardiology, University Clinic Giessen, Giessen, Germany; 13 Department of Cardiovascular Surgery, University Heart Centre Bad Krozingen, Freiburg, Germany; 14 Department of Cardiology, Koeln University Hospital, Koeln, Germany; 15 Department of Thorax and Cardiovascular Surgery, University Clinic Giessen, Giessen, Germany; 16 Department of Interventional Cardiology, Helios Klinikum Erfurt, Erfurt, Germany; 17 Department of Cardiothoracic Surgery, Charité - Universitätsmedizin Berlin Universität Berlin, Humboldt-Universität zu Berlin, and Berlin Institute of Health, Berlin, Germany; 18 Department of Cardiology, Heart Center Leipzig at University Leipzig and Leipzig Heart Institute, Leipzig, Germany; 19 II. Department of Medicine, University Medical Center Schleswig-Holstein, Lübeck, Germany

**Keywords:** Aortic stenosis, Mitral regurgitation, Paravalvular leak, TAVI, MSCT, Transthoracic echocardiogram

## Abstract

**Background:**

Patients with severe aortic stenosis (AS) frequently presented mitral regurgitation (MR), which may interfere with the standard echocardiographic measurements of mean pressure gradient (MPG), flow velocity, and aortic valve area (AVA).

**Aims:**

Herein we investigated the prevalence and severity of MR in patients with severe AS and its role on the accuracy of the standard echocardiographic parameters of AS quantification.

**Methods:**

Of all patients with severe AS undergoing transcatheter or surgical aortic valve replacement enrolled in the German Aortic Registry from 2011 to 2017, 119,641 were included in this study. The population was divided based on the values of left ventricular ejection fraction ([LVEF] > 50%, LVEF 31–50%, and LVEF ≤ 30%] and AVA (0.80 to ≤ 1.00 cm^2^, 0.60 to < 0.80 cm^2^, 0.40 to < 0.60 cm^2^, and 0.20 to < 0.40 cm^2^).

**Results:**

Overall, 77,890 (65%) patients with mild to-moderate and 4262 (4%) with severe MR were compared with 37,489 (31%) patients without MR. Patients with mild-to-moderate and severe MR presented significantly lower mPG (ΔmPG [95%CI] − 1.694 mmHg [− 2.123 to − 1.265], *p* < 0.0001 and − 6.954 mmHg [− 7.725 to − 6.183], *p* < 0.0001, respectively), that increased with LVEF impairment. Conversely, AVA did not differ (severe versus no MR: ΔAVA [95%CI]: − 0.007cm^2^ [− 0.023 to 0.009], *p* = 0.973). Increasing MR severity was associated with significant mPG reduction throughout all AVA strata, causing a low-gradient pattern, that manifested since the early stages of severe AS (LVEF > 50%: AVA 0.80 to 1.00 cm^2^; LVEF 31–50%: AVA 0.60 to 0.80 cm^2^).

**Conclusions:**

In patients with severe AS, concomitant MR is common, contributes to the onset of a low-gradient AS pattern, and affects the diagnostic accuracy of flow-dependent AVA measurements. In this setting, a multimodality, AVA-centric approach should be implemented.

**Graphical abstract:**

In patients with severe aortic stenosis, concomitant mitral regurgitation contributes to the onset of a low-gradient pattern, warranting a multimodality, and AVA-centric diagnostic approach.

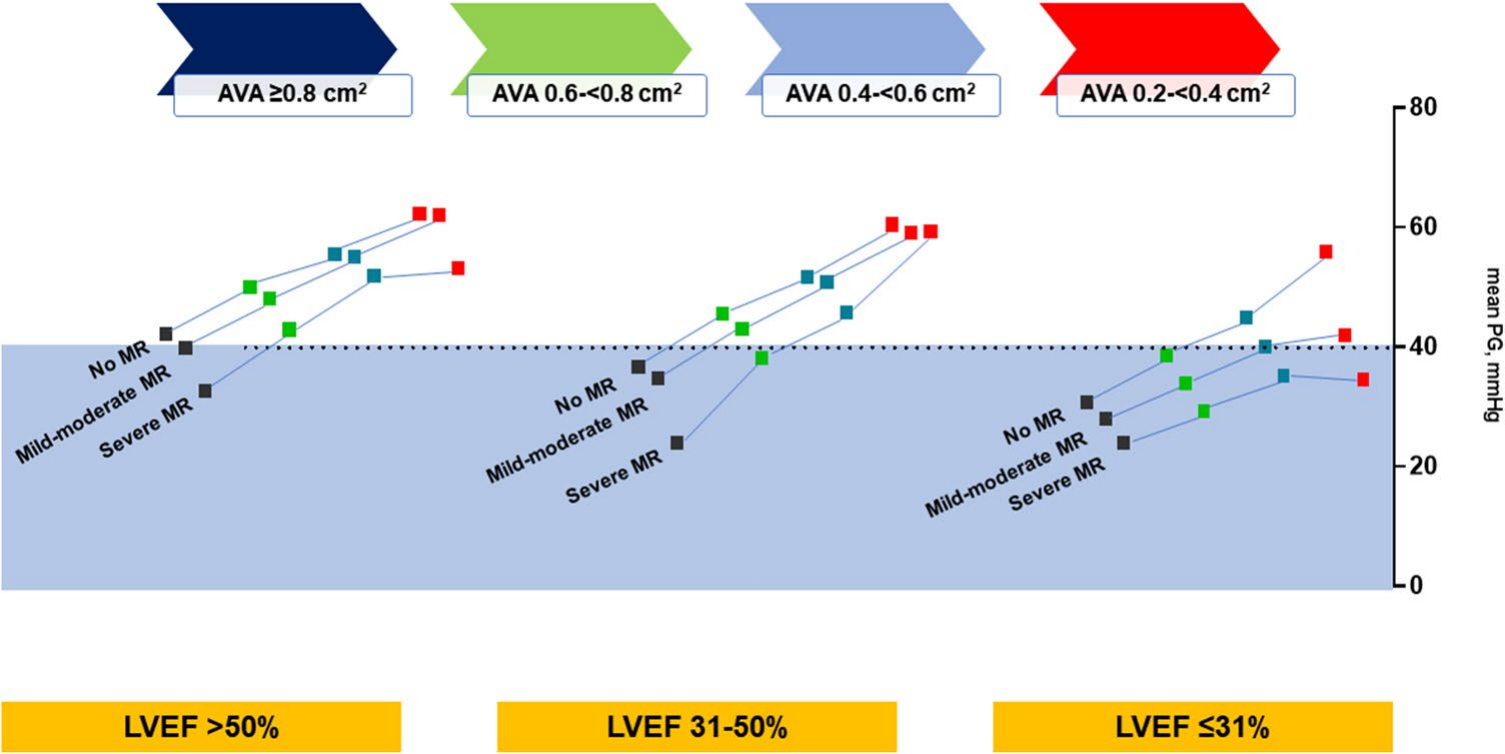

**Supplementary Information:**

The online version contains supplementary material available at 10.1007/s00392-022-02067-2.

## Introduction

Mitral regurgitation (MR) is a common coexisting finding present in up to two-thirds of patients with aortic stenosis (AS) [1] and approximately 20% of patients undergoing transcatheter (TAVR) or surgical aortic valve replacement (SAVR) have concomitant severe MR [2–4]. The coexistence of MR may cause a pattern of low-flow state with reduced stroke volume and valvular gradients, which can constitute a challenge in the confirmation of the true severe AS. Kate et al. demonstrated experimentally that relying on mean pressure gradient (MPG) or peak jet velocity (*V*max) underestimates the AS severity and that aortic valvular area (AVA) is the most reliable method to quantify AS severity [5].

MR may be caused directly by morphologic changes of the mitral valve or may be secondary to aortic stenosis and increased afterload and left ventricular dysfunction. In patients with severe AS, increased ventricular systolic pressure caused by aortic valve obstruction is a direct determinant of the degree of MR: the higher the left ventricular pressure, the more severe the MR for any given orifice area [6]. In patients with AS, MR is a major determinant of the low-flow condition responsible for the paradoxical low-flow low-gradient AS. Therefore, under these circumstances a careful quantitative assessment of both valves remains critical [7, 8]. Despite the frequent coexistence of AS and MR, the optimal treatment strategy of these patients remains a matter of debate, and guideline-based recommendations are lacking [9]. Careful patient evaluation is therefore crucial when trying to identify those who might profit from double valve interventions or re-evaluation of MR after surgical AVR or TAVR.

The aim of the present study was to investigate the prevalence and severity of MR in patients with severe AS from the German Aortic Valve Registry (GARY) undergoing isolated TAVR or SAVR from 2011 to 2017 and to assess the methodological limitations of the standard echocardiographic parameters of AS quantification in the presence of MR and left ventricular ejection fraction (LVEF) impairment.

## Methods

### Registry design

GARY is the largest, prospective, multicenter registry that monitors the safety and efficacy of interventional and surgical aortic valve procedures in Germany. Initiated in 2010, it is a registry of all comers with voluntary patient participation. The registry design has been described in detail previously [10]. In brief, data from 78 tertiary cardiovascular centers performing TAVR and/or SAVR were collected using standardized case report forms to record demographic, clinical, procedural, and follow-up data. The present analysis focuses on data of patients treated from 2011 to 2017. The investigators had full access to the data and control of the analysis. Initial approval for GARY was obtained from all participating centers, and patients gave written informed consent before the procedure.

### Study population

**Fig. 1:**
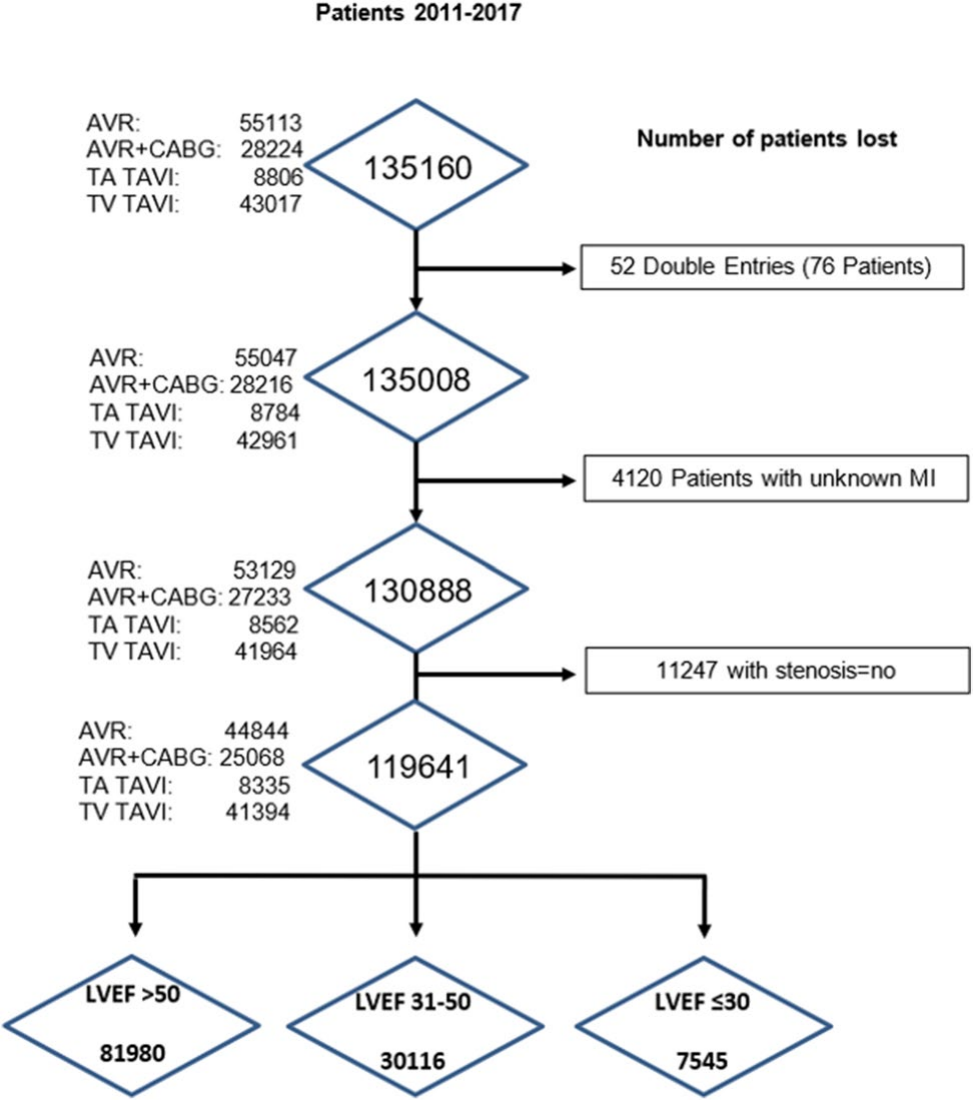
Flow chart of patient selection. The flowchart depicts the patient selection process from an initial population of patients being included in the German Aortic Valve Registry between 2011 and 2017. *AVR* aortic valve replacement, *CABG* coronary artery bypass graft, *LVEF* left ventricular ejection fraction, *MR* mitral regurgitation, *TAVI* transcatheter aortic valve intervention, *TV* transvascular, *TA* transapical

All patients undergoing aortic valve procedures for severe AS, either TAVR or SAVR, were eligible for inclusion. Of 135,160 patients enrolled between 2011 and 2017, 119,641 were included in the present study, after excluding double entries, those without AS and unknown cause of MR. Only patients with true severe AS and indication to treatment with either TAVR or SAVR were included in this registry. The study cohort was investigated for the presence and severity of MR and further divided into three groups based on the values of LVEF: group 1 (LVEF > 50%), group 2, (LVEF 31–50%), and group 3 (LVEF ≤ 30%). The decision to further stratify according to the value of LVEF was based on the known association with MR severity and its key role in the diagnostic algorithm of severe AS, as per guideline recommendations [11]. Figure 1 depicts the flow chart of the patient selection process.

### Quantification of AVA and MR and study endpoints

The severity of AS and MR in each center were estimated invasively with cardiac catheterization, and non-invasively with transthoracic (TTE) and transesophageal echocardiography, including 2- or 3-dimensional (2–3D) and Doppler imaging, as per guideline recommendations [12, 13]. For AS quantification, the following reported parameters were considered in the present study: maximal and mean pressure gradient (echocardiographically measured *P*max and *P*mean) and the invasive peak-to-peak gradient and AVA, calculated from the continuity equation in TTE or invasively with the Gorlin formula by using a Fick or thermodilution cardiac output measurement [14, 15]. All patients (119,641) had an echocardiographic quantification of AVA according to the continuity equation. Of these, 102,638 (86%) had a planimetric quantification and in 101,140 (85%) the Gorlin´s formula after invasive measurement of the peak-to-peak pressure gradient was used for AVA quantification. Severe AS was defined as AVA < 1.0 cm^2^ or indexed AVA < 0.6 cm^2^/m^2^ [11, 12]. Low flow was arbitrarily defined by a stroke volume index (SVi) ≤ 35 mL/m^2^, as per guideline recommendations [11]. The presence of a systolic pulmonary arterial pressure > 55 mmHg was defined as severe pulmonary hypertension (PH), as indicated in the routinely used perioperative risk scores and guideline recommendations [11].

In each center, after appropriate measurement, MR was quantified as none or trace, mild, moderate, or severe. For the present study, each LVEF group was further divided in three subgroups, based on the severity of MR: no MR (none or trace), mild-to-moderate MR, severe MR. The endpoints of the present study were changes in values of AVA and mPG according to the severity of MR and LVEF.

### Statistical analysis

The aim of this study was to analyze the association of MR severity with parameters of AS quantification, in an all-comers cohort of patients with severe AS.

For description of the MR severity quantification, mean ± standard deviation (SD) was shown for continuous variables and frequencies with percentages for categorical variables. Comparisons for the MR severity grades were performed with Kruskal–Wallis Tests for continuous variables and *χ*^2^ tests for categorical variables. To compare the difference of mPG and AVA according to MR severity in the three LVEF groups two-way ANOVA with interactions as well as nonparametric aligned rank ANOVA with interactions were performed.

Mean difference (Δ) with 95% confidence intervals [CI] were reported when comparing groups with different MR severity from two-way ANOVA and even more general ANOVA with interactions with significance corrections for multiple post hoc tests. For further investigating differences of mPG a subgroup analysis after stratifying for AVA was performed. A two-sided P value of ≤ 0.05 was considered statistically significant. Statistical analysis was performed with SAS statistical software (version 9.4, SAS Institute, Cary, NC, USA) and R (R Foundation for Statistical Computing, Vienna, Austria). The R packages ‘dplyr’ were used to perform a Kruskal–Wallis test, ‘ARTool’ and ‘emmeans’ for aligned rank ANOVA.

## Results

### Patient characteristics

**Fig. 2:**
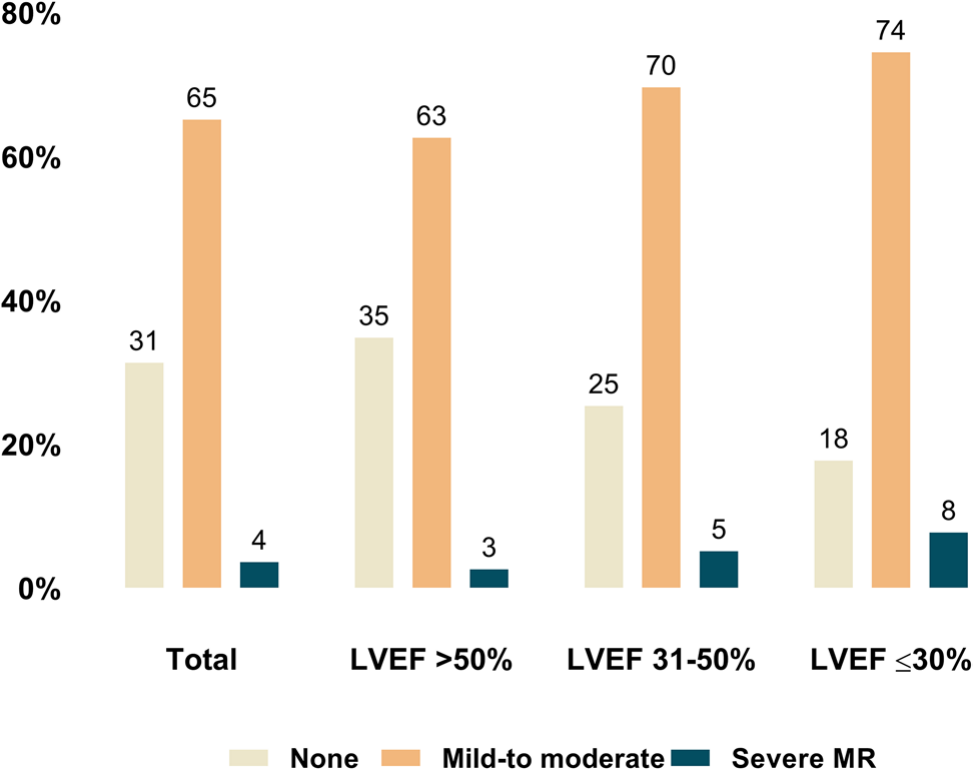
Prevalence of mitral regurgitation in patient with severe aortic stenosis. Prevalence of MR severity in patients with severe aortic stenosis, overall and according to LVEF impairment. *MR* mitral regurgitation, *LVEF* % of left ventricular ejection fraction, *MR I–II* mild-to-moderate MR, *MR III–IV* severe MR

Of 119,641 patients eligible for this study, 37,489 (31%) had no MR, whereas 77,890 (65%) and 4262 (4%) presented mild-to-moderate and severe MR, respectively (Fig. 2). Baseline characteristics of patients according to MR severity are shown in Table 1. In the overall population mean age was 75.02 ± 10 years and patients were predominantly male (57.6%). They had frequently hypertension (86%), diabetes (30%), a previous myocardial infarction (MI), percutaneous coronary intervention (20%), chronic heart failure (CHF, 25%), and atrial fibrillation (AF, 21%). At admission, patients were symptomatic (NYHA functional class II, III and IV: 25%, 63%, and 8%, respectively) and had an intermediate-high operative risk (mean EuroSCORE 13.9 ± 13.8%). Echocardiographically, severe pulmonary hypertension (PH) and tricuspid regurgitation (TR) were present in 11.1% and 2% of patients, respectively. Mean LVEF was 54.7 ± 12.6%, being normal (LVEF > 50%) or moderately (LVEF 31–50%) and severely (LVEF ≤ 30%) impaired in 69%, 25%, and 6% of patients, respectively.

**Table 1:** Baseline characteristics of patients according to MR severity

		Mitral regurgitation			
	All 119,641 (100%)	None 37,489 (31%)	Mild-to-moderate 77,890 (65%)	Severe 4262 (4%)	*P* value
Age, years	75.02 ± 10.00	71.42 ± 10.78	76.63 ± 9.17	77.36 ± 8.99	< 0.0001
Male	68,899 (57.6%)	23,970 (63.9%)	42,750 (54.9%)	2,179 (51.1%)	< 0.0001
Female	50,742 (42.4%)	13,519 (36.1%)	35,140 (45.1%)	2,083 (48.9%)	< 0.0001
BMI, kg/m^2^	27.9 ± 4.9	28.3 ± 4.9	27.7 ± 4.9	26.6 ± 4.7	< 0.0001
Hypertension	101,956 (85.8%)	31,134 (83.6%)	67,214 (86.9%)	3608 (85.6%)	< 0.0001
Diabetes	36,095 (30.2%)	10,281 (27.4%)	24,550 (31.5%)	1264 (29.7%)	< 0.0001
Previous PCI	23,742 (19.8%)	5547 (14.8%)	17,212 (22.1%)	983 (23.1%)	< 0.0001
Previous MI	14,190 (11.9%)	3283 (8.8%)	10,307 (13.3%)	600 (14.1%)	< 0.0001
CHF	29,657 (25.4%)	6043 (16.4%)	21,774 (28.7%)	1840 (44.6%)	< 0.0001
AF	25,570 (21.4%)	4824 (12.9%)	19,004 (24.4%)	1742 (40.9%)	< 0.0001
Heart surgery	13,155 (11.0%)	3110 (8.3%)	9342 (12.0%)	703 (16.5%)	< 0.0001
Lung disease	20,541 (17.2%)	5508 (14.7%)	14,185 (18.2%)	848 (19.9%)	< 0.0001
Clinical characteristics at admission					
Creatinine	1.10 ± 0.5	1.04 ± 0.4	1.12 ± 0.5	1.23 ± 0.5	< 0.0001
NYHA functional class					
II	29,529 (24.7%)	11,739 (31.3%)	17,167 (22.0%)	623 (14.6%)	< 0.0001
III	75,119 (62.8%)	21,217 (56.6%)	51,015 (65.5%)	2887 (67.7%)	< 0.0001
IV	9432 (7.9%)	2220 (5.9%)	6533 (8.4%)	679 (15.9%)	< 0.0001
EuroSCORE	13.9 ± 13.84	9.6 ± 10.4	15.5 ± 14.4	20.9 ± 17.8	< 0.0001
STS Score	4.1 ± 4.1	3.0 ± 3.0	4.5 ± 4.3	6.0 ± 5.9	< 0.0001
Echocardiographic parameters at admission					
PH > 55 mmHg	13,072 (11.1%)	1790 (4.9%)	10,083 (13.2%)	1199 (28.8%)	< 0.0001
Severe TR	2302 (2.0%)	193 (0.5%)	1501 (2.0%)	608 (14.7%)	< 0.0001
LVEF, %	54.7 ± 12.6	57.5 ± 11.3	53.8 ± 12.8	48.80 ± 14.3	< 0.0001
LVEF > 50%	81,980 (68.5%)	28,538 (76.1%)	51,297 (65.9%)	2145 (50.3%)	< 0.0001
LVEF 31–50%	30,116 (25.2%)	7612 (20.3%)	20,972 (26.9%)	1532 (35.9%)	< 0.0001
LVEF ≤ 30%	7545 (6.3%)	1339 (3.6%)	5621 (7.2%)	585 (13.7%)	< 0.0001
AV area, cm^2^	0.77 ± 0.4	0.80 ± 0.4	0.75 ± 0.3	0.79 ± 0.4	< 0.0001
AV-*P*mean, mmHg	44.1 ± 16.5	45.6 ± 16.1	43.8 ± 16.5	37.0 ± 17.3	< 0.0001
AV-*P*max, mmHg	70.4 ± 24.2	72.0 ± 23.6	70.2 ± 24.3	60.3 ± 26.1	< 0.0001

*AF* atrial fibrillation, *AV* aortic valve, *BMI* body mass index, *CHF* chronic heart failure, *MI* myocardial infarction, *MR* mitral regurgitation, *NYHA* New York Heart Association, *PCI* percutaneous coronary intervention, *Pmean* mean pressure gradient, *PH* pulmonary arterial hypertension, *STS* society of thoracic surgeons, *TR* tricuspid regurgitation

Patients with severe and mild-to-moderate MR were older and had more frequently comorbidities, including CHF and AF, compared to those without MR. At admission they presented significant perioperative symptoms (NYHA class III: 68%, 66%, and 57%; NYHA class IV: 16%, 8%, and 6%, respectively) and had higher operative risk (EuroSCORE: 20.9 ± 17.8, 15.5 ± 14.4, and 9.6 ± 10.4%), PH > 55 mmHg (29%, 13%, and 5%), and severe TR (15%, 2.0%, and 0.5%, respectively).

Patients with severe MR presented significantly more frequently with an LVEF < 30%, as compared to those with mild-to-moderate and no MR (14%, 7%, and 4%, respectively). With increasing MR severity patients had smaller AVAs and lower mPGs (AVA: 0.79 ± 0.4 cm^2^, 0.75 ± 0.3 cm^2^, and 0.80 ± 0.4 cm^2^, mPG: 37.0 ± 17.3 mmHg, 43.8 ± 16.5 mmHg, and 45.6 ± 16.1 mmHg in severe and mild-to-moderate versus no MR, respectively).

The above-described distribution pattern of characteristics in patients with severe, as compared to those with no MR, was also observed in the different LVEF impairment groups. Indeed, in each LVEF group, but especially in the severely impaired LVEF, patients with severe MR were older and presented more often comorbidities, prohibitive operative risk, AF, and aggravating symptoms (supplemental Tables 1–3).

### Hemodynamic profile of AVA and mPG in the presence of mitral regurgitation and LVEF impairment

**Fig. 3:**
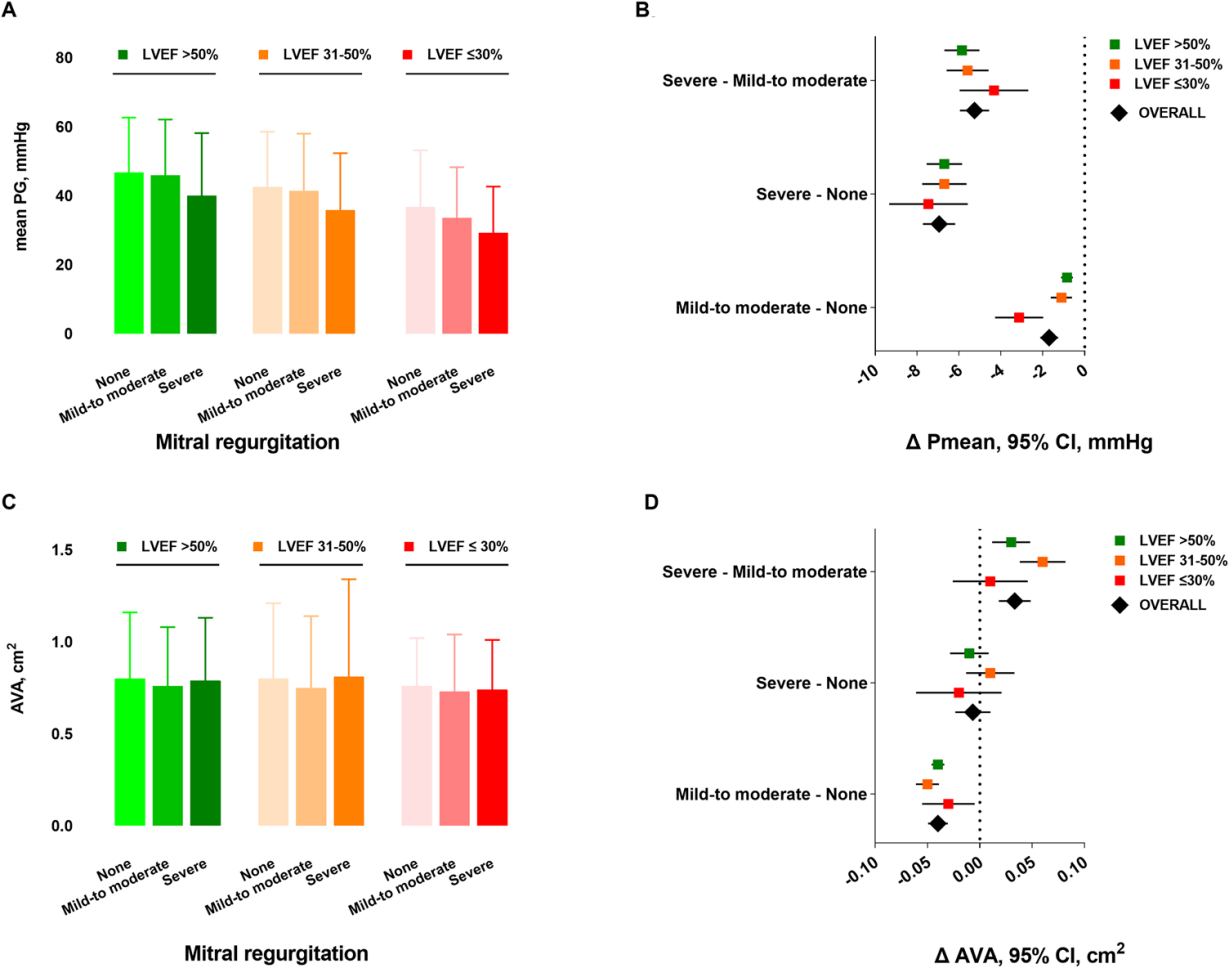
Changes of mPG and AVA in the presence of MR and LVEF impairment. Depicted are mean ± standard deviations (**A**, **C**) and mean differences with 95% confidence intervals (symbols with lines, **B**, **D**) calculated with two-way ANOVA for values of mPG (**A**–**B**) and AVA (**C**–**D**) according to MR severity overall (diamond symbol) and in each LVEF group (square symbol). *AVA* aortic valve area, *mPG* mean pressure gradient, *LVEF* % of left ventricular ejection fraction, *MR* mitral regurgitation

The quantification parameters of AS according to MR severity and within different LVEF groups are shown in Table 2. Overall, the values of AVA, mPG, and peak-to-peak gradient were significantly lower in the presence of severely impaired LVEF(< 30%), compared to LVEF 31–50% and LVEF > 50% (mean ± SD AVA: 0.73 ± 0.30 versus 0.76 ± 0.40 versus 0.77 ± 0.33 cm^2^, *p* < 0.0001; mPG: 33.74 ± 14.93 versus 41.4 ± 16.47 versus 46 ± 16.19 mmHg, *p* < 0.001; peak-to-peak gradient: 41.91 ± 20.23 versus 50.01 ± 23.17 versus 54.64 ± 22.92 mmHg, *p* < 0.0001). In each LVEF group a significant reduction of mPG in the presence of increasing MR severity was observed. Figure 3 depicts the mean values of mPG and AVA in the left panels (A, C), and the differences of mPG and AVA (Δ mPG and Δ AVA) in the right panels (B, D) according to different grades of MR severity, overall, and within each LVEF group.

**Table 2:** Parameters of AS quantification according to MR severity and LVEF

		Mitral regurgitation			
	All (*n* = 81,980)	None (*n* = 28,538)	Mild-to-moderate (*n* = 51,297)	Severe (*n* = 2145)	*p* value
*LVEF > 50%*					
AVA, cm^2^	0.77 ± 0.33	0.80 ± 0.36	0.76 ± 0.32	0.79 ± 0.34	< 0.0001
*P*max, mmHg	73.41 ± 23.55	73.84 ± 23.19	73.52 ± 23.56	64.86 ± 26.12	< 0.0001
*P*mean, mmHg	46.00 ± 16.19	46.71 ± 15.91	45.87 ± 16.20	40.01 ± 18.14	< 0.0001
Peak-to-peak, mmHg	54.64 ± 22.92	56.71 ± 22.29	54.06 ± 23.07	50.05 ± 23.33	< 0.0001

		Mitral regurgitation			
	All (*n* = 30,116)	None (*n* = 7612)	Mild-to-moderate (*n* = 20,972)	Severe (*n* = 1532)	*p* value
*LVEF 31–50%*					
AVA, cm^2^	0.76 ± 0.40	0.80 ± 0.41	0.74 ± 0.34	0.81 ± 0.53	< 0.0001
Pmax, mmHg	66.03 ± 24.21	67.17 ± 23.50	66.20 ± 24.21	58.36 ± 25.96	< 0.0001
Pmean, mmHg	41.40 ± 16.47	42.54 ± 15.97	41.43 ± 16.55	35.84 ± 16.47	< 0.0001
Peak-to-peak, mmHg	50.01 ± 23.17	51.62 ± 22.61	49.99 ± 23.28	45.08 ± 22.94	< 0.0001

		Mitral regurgitation			
	All (*n* = 7545)	None (*n* = 1339)	Mild-to-moderate (*n* = 5621)	Severe (*n* = 585)	*p* value
*LVEF ≤ 30%*					
AVA, cm^2^	0.73 ± 0.30	0.76 ± 0.26	0.73 ± 0.31	0.74 ± 0.27	0.0006
*P*max, mmHg	54.37 ± 22.47	57.71 ± 23.52	54.33 ± 22.18	48.39 ± 22.06	< 0.0001
*P*mean, mmHg	33.74 ± 14.93	36.73 ± 16.42	33.60 ± 14.65	29.27 ± 13.36	< 0.0001
Peak-to-peak, mmHg	41.91 ± 20.23	45.56 ± 21.80	41.68 ± 19.89	38.39 ± 20.21	0.0027

*AVA* aortic valve area, *LVEF* % of left ventricular ejection fraction, *Pmax* maximal pressure gradient, *Pmean* mean pressure gradient, *Peak-to-peak* maximal pressure gradient measured invasively, *MR* mitral regurgitation

*P* values are from Kruskal–Wallis Test

Multiple comparison analysis showed that with increasing MR severity values of mPG lowered significantly overall and within the LVEF groups (Fig. 3B). Indeed, compared to patients without MR, those with mild-to-moderate and severe MR had a significant, increasing difference of mPG (ΔmPG [95%CI] − 1.694 mmHg [− 2.123 to − 1.265], *p* < 0.0001 and − 6.954 mmHg [− 7.725 to − 6.183], *p* < 0.0001, respectively), that increased further with worsening of the LVEF function (supplemental Table 4). Overall, this pattern was confirmed in an ANOVA analysis adjusting for age, sex, body mass index, and atrial fibrillation (supplemental Table 5).

The distribution pattern of AVA in patients with and without MR and within the LVEF groups is depicted in Fig. 3D and supplemental Table 6. Patients with mild–moderate but not those with severe MR had significantly smaller AVA compared to those without MR (ΔAVA [95%CI] − 0.039cm^2^ [− 0.049 to − 0.031], *p* < 0.0001, and − 0.007cm^2^ [− 0.023 to 0.009], *p* = 0.976, respectively). Interestingly, patients with severe MR had more frequently larger AVA compared to those with mild-to-moderate MR (ΔAVA [95%CI]: 0.033 cm^2^ [0.018 to 0.047], *p* < 0.0001). This distribution pattern of AVA was maintained in the different LVEF groups (supplemental Table 6) and, overall, also confirmed in an ANOVA analysis adjusting for age, sex, body mass index, and atrial fibrillation.

### AVA subgroup and interaction analysis

**Fig. 4:**
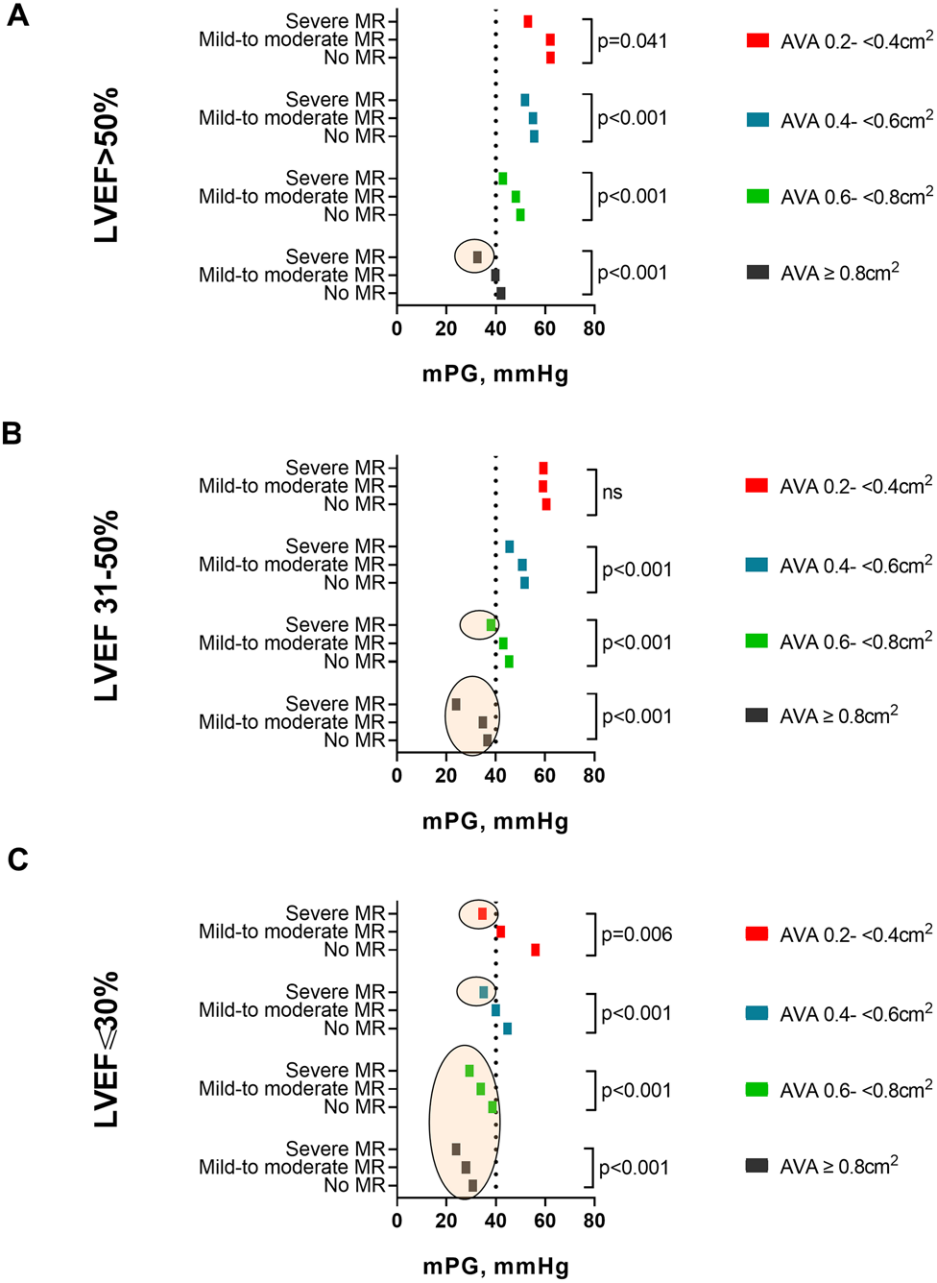
Hemodynamic profile of mPG, MR, and LVEF within different AVA strata. The figure depicts the differences in values of mean mPG according to MR severity in different AVA strata for patients with **A** LVEF > 50%, **B** LVEF 31–50%, and **C** LVEF ≤ 30%. The colored circles highlight cases with mPG < 40 mmHg. *P* values are from Kruskal–Wallis tests in the respective subgroups. There is a significant interaction between AVA and MR severity on mPG. *AVA* aortic valve area in cm^2^, *mPG* mean pressure gradient, *LVEF* % of left ventricular ejection fraction, *MR* mitral regurgitation, *ns* non-significant

After stratifying the population in different AVA strata, (AVA 0.8 to 1.0 cm^2^, 0.6 to < 0.8 cm^2^, 0.4 to < 0.6 cm^2^ and 0.2 to < 0.4 cm^2^) the values of mPG were analyzed in the presence of increasing MR severity within the LVEF groups (Fig. 4). At baseline (no MR), patients with LVEF > 50% and LVEF 31–50% had high valvular gradients (mPG ≥ 40 mmHg), that remained above 40 mmHg in all AVA strata, except for AVA 0.8 to 1.0 cm^2^, despite a significant reduction with increasing MR severity (Fig. 4A–B). Conversely, patients with severely impaired LVEF (≤ 30%) had a low baseline gradient (mPG < 40 mmHg) in all AVA strata, except in the presence of smaller valvular areas (AVA < 06 cm^2^, Fig. 4C).

With MR progression, patients displayed a significant reduction of mPG throughout all AVA strata within each LVEF group, except for patients with LVEF 31–50% and with exceedingly small valvular areas (AVA 0.2 to < 0.4 cm^2^ Fig. 4B). With two-fold aligned rank ANOVA for mPG, the interaction between MR severity and LVEF was significant in the largest group (AVA between 0.8 and 1.0 cm^2^) and overall (*p* = 0.0106 and *p* = 0.0053, respectively).

Patients with LVEF > 50% manifested a low-gradient pattern only in the early AS disease stages, with lager valvular areas (AVA 0.8 to 1.0 cm^2^), and concomitant presence of severe MR (Fig. 4A, colored circle). Instead, patients with LVEF 31–50% presented an MR-independent low gradient for AVA 0.8 to 1.0 cm^2^, whereas in the later stages of AS (AVA 0.6 to < 0.8 cm^2^) it occurred only in the presence of severe MR (Fig. 4B, colored circles). In patients with severely impaired LVEF (< 30%), a low valvular gradient (mPG < 40 mmHg) was common since the early disease stages, characterized from large valvular areas (AVA ≥ 0.6 cm^2^), independently from the presence of concomitant MR, whereas in the later stages, with AVA reduction (< 0.6 cm^2^), it occurred only in the presence of severe MR (Fig. 4C, colored circles).

## Discussion

This is the first GARY study investigating the effect of concomitant MR on parameters of AS quantification in patients undergoing TAVR or SAVR for severe AS. In this large cohort of 119,641 patients, we found that the following:

1. In patients with severe AS, irrespectively of the LVEF function, there is a high prevalence of MR (69%), whose severity MR increases with the degree of LVEF impairment (Fig. 2).
2. The main parameters of AS quantification, mPG and AVA, behave differently in the presence of MR: whereas mPG reduces with increasing MR severity and LVEF impairment, values of AVA are similarly distributed in patients with severe versus those without MR (Fig. 3).
3. There is a significant interaction between the severity of MR, LVEF impairment, and AVA in terms of mPG, which lowers in the presence of larger AVA, impaired LVEF, and increasing MR severity (Fig. 4). The increasing MR severity causes a low-gradient pattern that, especially in the initial phases of severe AS and absence of severely impaired LVEF, could delay the diagnosis and early treatment referral.
4. The timely progression of AS with consequent AVA narrowing causes an increase of mPG, whereas the progression of MR has the opposite effect. The effect of both these factors on the mPG differs in magnitude according to the degree of LVEF impairment at the initial phases of AS disease process (Fig. 4).

Concomitant MR in patients with severe AS is common and is reportedly associated to high early and late mortality after TAVR or SAVR [16–20]. In line with previously reported data from a large meta-analysis of > 8000 patients [16], in the present study we report, in a large population of 119641 patients with severe AS, a prevalence of 65% and 4% for mild-to-moderate and severe MR, respectively. With progressive LVEF impairment we observed an increase in the prevalence of severe MR, from 3 to 8% in patients with LVEF > 50% and ≤ 30%, respectively. These were all symptomatic patients, with a prohibitive perioperative risk, as demonstrated by the presence of several comorbidities and the high value of the perioperative risk scores.

The coexistence of MR in patients with severe AS poses a challenge regarding the standard echocardiographic assessment and quantitative graduation of both valvular pathologies. In the presence of MR, the increased LV afterload related to AS, combined with MR, results in a decrease in forward LV stroke volume and transvalvular gradient, and therefore, often in a low-flow, low-gradient pattern, underestimating the severity of AS [21]. Our data show a significant interaction between MR severity, values of AVA and mPG within the different groups of LVEF impairment. We demonstrate a significant reduction of mPG with increasing MR severity and LVEF impairment and show for the first time that this pattern of mPG reduction is maintained within the different AVA strata, which represent a timely progression of the AS (Fig. 4). The progression of AS, as supposed from decreasing AVA, causes an increase of mPG, whereas the concomitant progression of MR reduces it by reducing the stroke volume. Under these conditions the net mPG depends on the degree of remodeling of LV and its contribution to the final stroke volume, hence, the lower mPG values for impaired LVEF.

We observed that in the presence of LV dysfunction, concomitant severe MR triggers the manifestation of a low-gradient pattern of AS for specific values of AVA. In fact, the presence of severe MR can mask an underlying severe AS in patients with LVEF < 30% so that mPG is always low or until the disease progresses to lower AVA values causing the shifting of mPG above 40 mmHg. The cardiologic community is already sensitive to the problem of low-gradient AS in patients with severely impaired LVEF, who since some time now are being carefully evaluated, as indicated in guideline recommendations [9]. On the other hand, for the more complex patients with multivalvular disease, there is a general lack of data on natural history of mixed valvular disease that support the early diagnosis and final management decision.

In our cohort of patients with AS and concomitant MR we observed that the presence of severe MR can mask a severe AS in patients with LVEF > 50% and AVA 0.8 to 1.0 cm^2^ or LVEF 31–50% and AVA 0.6 to < 0.8 cm^2^ by causing a low-gradient pattern. (Fig. 4A–B). Considering the smaller AVAs characterize a later stage of the natural history of AS, the low-gradient pattern present in the early stages of severe AS could delay the appropriate diagnosis and treatment. Therefore, patients with MR and AS should be very carefully evaluated for the presence of severe AS to avoid poor outcomes [22, 23].

Although both AS and MR can, in the long term, induce LV negative remodeling and dysfunction, the early detection of LV dysfunction in these patients with the current standard assessment methods is impeded. Therefore, a comprehensive integrated approach using quantitative and semiquantitative echocardiographic parameters, or other imaging modalities, such as cardiac computed tomography or magnetic resonance (CMR), is particularly important since the early stages of a bivalvular AS/MR [13]. Furthermore, in case of equivocal parameters with the abovementioned approaches, additional parameters are used for the assessment of severe AS, such as the resting Doppler velocity index—does not require calculation of LVOT area and CCT [24]. The latter approach may be especially useful when combined with geometric assessment of valve area in assessing the severity of AS in patients with low valve gradient [25]. As result of elevated LV filling pressures generated in the presence of severe AS, the severity of concomitant MR may be overestimated, therefore a careful quantification is required. Recently, Benfari et al. showed that in the presence of severe AS, the effective regurgitant orifice area (EROA) is the most reliable parameter of MR quantification and predicts the presence of low flow [26]. Therefore, all patients with severely impaired LVEF and any degree of MR should be carefully assessed for the presence of low-gradient severe AS.

As indicated from our data, patients with moderately impaired or normal LVEF and low-gradient AS due to concomitant mild-to-moderate and severe MR should also be carefully assessed, since underestimating AS severity, especially in the early disease stages when AVA is still relatively large (AVA 0.8 to 1cm^2^), increases mortality due to late referral [27]. Under these circumstances, mPG is not a reliable parameter for the assessment of true severe AS; therefore, a multimodality approach combining, for example, 3D echocardiography and deformation analysis of the LV and CMR, should be considered [28, 29].

In summary, the effect of coexisting MR and severe AS depends on a complex interplay of pathophysiological factors, including the severity of each individual valve lesion, grade of ventricular remodeling, and function impairment. In the setting of MR, a subtype of low-flow low-gradient AS generates; therefore, flow-dependent parameters as mPG and Vmax are not reliable, instead, a multimodality AVA-centric approach should be used for the early detection and referral of true severe AS.

### Limitations

This study is limited by its non-randomized design, being a large, prospective, and all-comers registry. Secondly, no detailed information regarding the quantification methods for MR was available. However, as mentioned above, this registry has the advantage of permitting large-scale data analysis and real-world, all-comers data collection. No core lab adjudication of echocardiographic data and no temporal follow-up of the progression of AS was available. Nonetheless, by dividing AVA at the time of diagnosis of severe AS, in four strata we assumed that in the earlier disease stages AVA is larger, and with disease progression becomes narrower. No outcome data after TAVI were available for further analysis; nonetheless, the negative association of concomitant MR in patients with severe AS has already been demonstrated in several published reports [18, 23]. Data were obtained with TAVR techniques used during the years 2011 to 2017; thus, further analyses will be necessary in the coming years to address new technical achievements.

## Conclusions

In patients with severe AS, the presence of concomitant MR is common and limits the diagnostic accuracy of standard echocardiographic AS quantification. Increasing MR severity reduces the transvalvular aortic mPG, resulting in a low-gradient pattern, that especially in the initial phases of severe AS and absence of severely impaired LVEF, can delay the diagnosis and early treatment referral. Therefore, in the presence of bivalvular disease a multimodality AVA-centric approach should be used for the early detection of true severe AS.

## Supplementary Information

Below is the link to the electronic supplementary material.

Supplementary file1 (DOCX 47 KB)
