# Supplementary material for: Concomitant mitral regurgitation in patients with low-gradient aortic stenosis: an analysis from the German Aortic Valve Registry
Source: Clin Res Cardiol. 2022 Aug 19;111(12):1377–86. doi: 10.1007/s00392-022-02067-2 (PMC9681685; doi:10.1007/s00392-022-02067-2)
Supplement: Supplementary file 1 — Supplementary file1 (DOCX 47 KB) [file 392_2022_2067_MOESM1_ESM.docx]

**Low-gradient aortic stenosis in patients with concomitant mitral regurgitation – an analysis from the German Aortic Valve Registry (GARY)**

## SUPPLEMENTAL MATERIAL

## Supplemental Table 1. Baseline characteristics of patients with LVEF > 50 %

|  | **All**  **81,980** | **No MR**  **28,538 (35%)** | **Mild-moderate MR**  **51,297 (63%)** | **Severe MR**  **2,145 (3%)** | **p value** |
| --- | --- | --- | --- | --- | --- |
| **Age (years)** | 74.57 ± 10.16 | 70.88 ± 10.87 | 76.50 ± 9.17 | 77.58 ± 9.26 | <0.0001 |
| **Male** | 44,126 (53.8 %) | 17,627 (61.8 %) | 25,550 (49.8 %) | 949 (44.2 %) | <0.0001 |
| **Female** | 37,854 (46.2 %) | 10,911 (38.2 %) | 25,747 (50.2 %) | 1,196 (55.8 %) | <0.0001 |
| **BMI (kg/m²)** | 28.03 ± 4.87 | 28.43 ± 4.86 | 27.86 ± 4.86 | 26.68 ± 4.75 | <0.0001 |
| **Hypertension** | 69,825 (85.7 %) | 23,656 (83.4 %) | 44,353 (87.0 %) | 1816 (85.5 %) | <0.0001 |
| **Diabetes** | 23,078 (28.2 %) | 7,408 (26.0 %) | 15,120 (29.5 %) | 550 (25.7 %) | <0.0001 |
| **Previous PCI** | 14,010 (17.1 %) | 3,710 (13.0 %) | 9,879 (19.3 %) | 421 (19.6 %) | <0.0001 |
| **Previous MI** | 6,971 (8.5 %) | 1,910 (6.7 %) | 4,867 (9.5 %) | 194 (9.1 %) | <0.0001 |
| **CHF** | 15,187 (19.0 %) | 3,408 (12.2 %) | 10,989 (22.0 %) | 790 (38.0 %) | <0.0001 |
| **Atrial fibrillation** | 14,642 (17.9 %) | 2,964 (10.4 %) | 10,812 (21.1 %) | 866 (40.4 %) | <0.0001 |
| **Previous cardiac surgery** | 7,268 (8.9 %) | 1,978 (6.9 %) | 5,011 (9.8 %) | 279 (13.0 %) | <0.0001 |
| **Lung disease** | 12,780 (15.6 %) | 3,868 (13.6 %) | 8,495 (16.6 %) | 417 (19.5 %) | <0.0001 |
| **Clinical And Echocardiographic Characteristics At Admission** | | | | | |
| **Creatinine** | 1.05 ± 0.43 | 1.01 ± 0.39 | 1.07 ± 0.44 | 1.17 ± 0.50 | <0.0001 |
| **NYHA class** |  |  |  |  | <0.0001 |
| **I** | 4,563 (5.6 %) | 1,997 (7.0 %) | 2,517 (4.9 %) | 49 (2.3 %) |  |
| **II** | 23,065 (28.1 %) | 9,742 (34.1 %) | 12,939 (25.2 %) | 384 (17.9 %) |  |
| **III** | 49,684 (60.6 %) | 15,425 (54.1 %) | 32,797 (63.9 %) | 1,462 (68.2 %) |  |
| **IV** | 4668 (5.7 %) | 1374 (4.8 %) | 3044 (5.9 %) | 250 (11.7 %) |  |
| **Euro-Score (%)** | 10.55 ± 10.27 | 7.68 ± 7.87 | 11.95 ± 10.91 | 15.46 ± 13.52 | <0.0001 |
| **STS-Score (%)** | 3.56 ± 3.37 | 2.65 ± 2.56 | 3.99 ± 3.57 | 5.30 ± 4.96 | <0.0001 |
| **PH >55 mmHg** | 6,949 (8.6 %) | 1,001 (3.6 %) | 5,363 (10.6 %) | 585 (28.0 %) | <0.0001 |
| **Severe TR** | 1,153 (1.4 %) | 109 (0.4 %) | 764 (1.5 %) | 280 (13.5 %) | <0.0001 |

Legend: AV-aortic valve, BMI-body mass index, CHF-chronic heart failure, MI-myocardial infarction, NYHA-New York Heart Association, PCI-percutaneous coronary intervention, Pmean-mean pressure gradient PH-pulmonary hypertension, STS-society of thoracic surgeons; TR: tricuspid regurgitation.

## Supplemental Table 2. Baseline characteristics of patients with LVEF 31-50 %

|  | **All**  **30116** | **No MR**  **7,612 (25%)** | **Mild-moderate MR**  **20,972 (70%)** | **Severe MR**  **1,532 (5%)** | **p value** |
| --- | --- | --- | --- | --- | --- |
| **Age (years)** | 76.22 ± 9.46 | 73.37 ± 10.22 | 77.16 ± 9.02 | 77.37 ± 8.55 | <0.0001 |
| **Male** | 19,261 (64.0 %) | 5,304 (69.7 %) | 13,117 (62.5 %) | 840 (54.8 %) | <0.0001 |
| **Female** | 10,855 (36.0 %) | 2,308 (30.3 %) | 7,855 (37.5 %) | 692 (45.2 %) | <0.0001 |
| **BMI (kg/m²),** | 27.74 ± 4.90 | 28.23 ± 4.87 | 27.64 ± 4.91 | 26.67 ± 4.69 | <0.0001 |
| **Hypertension** | 25,883 (86.7 %) | 6,394 (84.8 %) | 18,165 (87.3 %) | 1,324 (87.3 %) | <0.0001 |
| **Diabetes** | 10,350 (34.4 %) | 2,451 (32.2 %) | 7,389 (35.3 %) | 510 (33.4 %) | <0.0001 |
| **Previous MI** | 5,378 (17.9 %) | 1,120 (14.8 %) | 3999 (19.1 %) | 259 (17.0 %) | <0.0001 |
| **Previous PCI** | 7,566 (25.1 %) | 1,540 (20.2 %) | 5,652 (27.0 %) | 374 (24.4 %) | <0.0001 |
| **CHF** | 10,568 (35.9 %) | 2,092 (27.9 %) | 7,766 (38.0 %) | 710 (47.7 %) | <0.0001 |
| **Atrial fibrillation** | 8,589 (28.5 %) | 1,522 (20.0 %) | 6,409 (30.6 %) | 658 (43.0 %) | <0.0001 |
| **Previous cardiac surgery** | 4,473 (14.9 %) | 919 (12.1 %) | 3,276 (15.6 %) | 278 (18.2 %) | <0.0001 |
| **Lung disease** | 6,092 (20.2 %) | 1,385 (18.2 %) | 4,400 (21.0 %) | 307 (20.1 %) | <0.0001 |
| **Clinical And Echocardiographic Characteristics At Admission** | | | | | |
| **Creatinine** | 1.17 ± 0.49 | 1.11 ± 0.44 | 1.19 ± 0.50 | 1.26 ± 0.55 | <0.0001 |
| **NYHA class** |  |  |  |  | <0.0001 |
| **I** | 858 (2.8 %) | 278 (3.7 %) | 561 (2.7 %) | 19 (1.2 %) |  |
| **II** | 5,651 (18.8 %) | 1,791 (23.5 %) | 3,668 (17.5 %) | 192 (12.5 %) |  |
| **III** | 20,415 (67.8 %) | 4,920 (64.6 %) | 14,441 (68.9 %) | 1,054 (68.8 %) |  |
| **IV** | 3,192 (10.6 %) | 623 (8.2 %) | 2302 (11.0 %) | 267 (17.4 %) |  |
| **Euro-Score (%)** | 18.64 ± 15.23 | 14.32 ± 12.89 | 19.91 ± 15.53 | 22.60 ± 17.47 | <0.0001 |
| **STS-Score (%)** | 4.86 ± 4.58 | 3.74 ± 3.76 | 5.17 ± 4.67 | 6.16 ± 5.80 | <0.0001 |
| **PH >55mmHg** | 4,432 (15.0 %) | 596 (8.0 %) | 3,401 (16.5 %) | 435 (28.9 %) | <0.0001 |
| **Severe TR** | 820 (2.8 %) | 69 (0.9 %) | 527 (2.6 %) | 224 (15.0 %) | <0.0001 |

Abbreviations as in Table 1.

## Supplemental Table 3. Baseline characteristics of patients with LVEF ≤ 30%

|  | **All**  **7,545 (100%)** | **No MR**  **1,339 (18%)** | **Mild-moderate MR**  **5,621 (74%)** | **Severe MR**  **585 (8%)** | **p value** |
| --- | --- | --- | --- | --- | --- |
| **Age (years)** | 75.16 ± 9.91 | 71.98 ± 10.84 | 75.77 ± 9.61 | 76.56 ± 9.10 | <0.0001 |
| **Male** | 5,512 (73.1 %) | 1,039 (77.6 %) | 4,083 (72.6 %) | 390 (66.7 %) | <0.0001 |
| **Female** | 2,033 (26.9 %) | 300 (22.4 %) | 1,538 (27.4 %) | 195 (33.3 %) | <0.0001 |
| **BMI (kg/m²)** | 26.96 ± 4.80 | 27.45 ± 4.86 | 26.92 ± 4.79 | 26.18 ± 4.61 | <0.0001 |
| **Hypertension** | 6,248 (83.9 %) | 1,084 (82.0 %) | 4,696 (84.6 %) | 468 (81.2 %) | 0.0128 |
| **Diabetes** | 2,667 (35.4 %) | 422 (31.6 %) | 2041 (36.3 %) | 204 (34.9 %) | 0.0049 |
| **Previous MI** | 1,841 (24.5 %) | 253 (19.0 %) | 1,441 (25.7 %) | 147 (25.3 %) | <0.0001 |
| **Previous PCI** | 2,166 (28.7 %) | 297 (22.2 %) | 1,681 (29.9 %) | 188 (32.1 %) | <0.0001 |
| **CHF** | 3,902 (53.2 %) | 543 (41.5 %) | 3,019 (55.3 %) | 340 (60.3 %) | <0.0001 |
| **Atrial fibrillation** | 2,339 (31.0 %) | 338 (25.2 %) | 1,783 (31.7 %) | 218 (37.3 %) | <0.0001 |
| **Previous cardiac surgery** | 1,414 (18.8 %) | 213 (16.0 %) | 1,055 (18.8 %) | 146 (25.0 %) | <0.0001 |
| **Lung disease** | 1,669 (22.1 %) | 255 (19.1 %) | 1,290 (23.0 %) | 124 (21.2 %) | 0.007 |
| **Clinical And Echocardiographic Characteristics At Admission** | | | | | |
| **Creatinine** | 1.29 ± 0.56 | 1.21 ± 0.48 | 1.31 ± 0.58 | 1.35 ± 0.58 | <0.0001 |
| **NYHA class** |  |  |  |  | <0.0001 |
| **I** | 140 (1.9 %) | 38 (2.8 %) | 97 (1.7 %) | 5 (0.9 %) |  |
| **II** | 813 (10.8 %) | 206 (15.4 %) | 560 (10.0 %) | 47 (8.0 %) |  |
| **III** | 5,020 (66.5 %) | 872 (65.1 %) | 3,777 (67.2 %) | 371 (63.4 %) |  |
| **IV** | 1,572 (20.8 %) | 223 (16.7 %) | 1,187 (21.1 %) | 162 (27.7 %) |  |
| **Euro-Score (%)** | 31.25 ± 20.71 | 24.88 ± 19.04 | 32.20 ± 20.62 | 36.50 ± 22.06 | <0.0001 |
| **STS-Score (%)** | 6.53 ± 6.59 | 4.83 ± 5.24 | 6.76 ± 6.62 | 8.19 ± 8.12 | <0.0001 |
| **PH > 55 mmHg** | 1,691 (22.9 %) | 193 (14.8 %) | 1,319 (24.0 %) | 179 (31.2 %) | <0.0001 |
| **Severe TR** | 329 (4.5 %) | 15 (1.1 %) | 210 (3.8 %) | 104 (18.4 %) | <0.0001 |

Abbreviations as in Table 1.

## Supplemental Table 4. Changes of ΔmPG in presence of MR

| **Comparison groups** | **Mean difference** | **95,00% CI** | **P Value** |
| --- | --- | --- | --- |
| **Overall** |  |  |  |
| Mild-to moderate vs. None | -1.694 | -2.123 to -1.265 | <0.0001 |
| Severe vs. None | -6.954 | -7.725 to -6.183 | <0.0001 |
| Severe vs. Mild-to moderate | -5.260 | -5.959 to -4.561 | <0.0001 |
| **LVEF>50% (group 1)** |  |  |  |
| Mild-to moderate vs. None | -0.837 | -1.116 to -0.558 | <0.0001 |
| Severe vs. None | -6.697 | -7.544 to -5.850 | <0.0001 |
| Severe vs. Mild-to moderate | -5.860 | -6.694 to -5.026 | <0.0001 |
| **LVEF 31-50% (group 2)** |  |  |  |
| Mild-to moderate vs. None | -1.116 | -1.622 to -0.610 | <0.0001 |
| Severe vs. None | -6.701 | -7.760 to -5.641 | <0.0001 |
| Severe vs. Mild-to moderate | -5.584 | -6.594 to -5.026 | <0.0001 |
| **LVEF≤30% (group 3)** |  |  |  |
| Mild-to moderate vs. None | -3.128 | -4.279 to -1.978 | <0.0001 |
| Severe vs. None | -7.465 | -9.339 to -5.590 | <0.0001 |
| Severe vs. Mild-to moderate | -4.336 | -5.980 to -2.693 | <0.0001 |

The results are from two way ANOVA with Tukey's multiple comparisons tests and significance results coincide with results from nonparametric aligned rank ANOVA with Tukey’s multiple comparisons tests with significance corrections. CI- confidence intervals, MR-mitral regurgitation, other abbreviations as in Table 1.

## Supplemental Table 5. Changes of ΔmPG in presence of MR adjusted for confounders

| **Comparison groups** | **Mean difference** | **95,00% CI** | **P Value** |
| --- | --- | --- | --- |
| **LVEF>50% (group 1)** |  |  |  |
| Mild-to moderate vs. None | -0.491 | -0.860 to -0.123 | 0.0024 |
| Severe vs. None | -5.682 | -6.802 to -4.562 | <0.0001 |
| Severe vs. Mild-to moderate | -5.191 | -6.284 to -4.097 | <0.0001 |
| **LVEF 31-50% (group 2)** |  |  |  |
| Mild-to moderate vs. None | -0.657 | -1.339 to 0.024 | 0.0657 |
| Severe vs. None | -5.352 | -6.769 to -3.935 | <0.0001 |
| Severe vs. Mild-to moderate | -4.694 | -6.020 to -3.369 | <0.0001 |
| **LVEF≤30% (group 3)** |  |  |  |
| Mild-to moderate vs. None | -2.906 | -4.498 to -1.314 | <0.0001 |
| Severe vs. None | -6.815 | -9.360 to -4.270 | <0.0001 |
| Severe vs. Mild-to moderate | -3.909 | -6.095 to -1.722 | <0.0001 |

Significance results are from ANOVA with the listed multiple comparisons tests using also adjustments by sex, age (<74, 75-79, 80-84, > 85 years), body mass index (≤25 vs. > 25 kg/m²), CI-confidence intervals, MR-mitral regurgitation, other abbreviations as in Table 1.

## Supplemental Table 6. Changes of ΔAVA in presence of MR

| **Comparison groups** | **Mean difference** | **95,00% CI ^1^** | **P Value^1^** |
| --- | --- | --- | --- |
| **Overall** |  |  |  |
| Mild-to moderate vs. None | -0.039 | -0.049 to -0.031 | <0.0001 |
| Severe vs. None | -0.0069 | -0.023 to 0.009 | 0.976 |
| Severe vs. Mild-to moderate | 0.033 | 0.018 to 0.047 | <0.0001 |
| **LVEF>50% (group 1)** |  |  |  |
| Mild-to moderate vs. None | -0.017 | -0.024 to -0.009 | <0.0001 |
| Severe vs. None | 0.029 | 0.006 to 0.052 | 0.0064 |
| Severe vs. Mild-to moderate | 0.046 | 0.023 to 0.069 | <0.0001 |
| **LVEF 31-50% (group 2)** |  |  |  |
| Mild-to moderate vs. None | -0.052 | -0.066 to -0.038 | <0.0001 |
| Severe vs. None | 0.017 | -0.013 to 0.047 | 0.017 |
| Severe vs. Mild-to moderate | 0.069 | 0.041 to 0.097 | <0.0001 |
| **LVEF≤30% (group 3)** |  |  |  |
| Mild-to moderate vs. None | -0.019 | -0.052 to 0.013 | 0.0001 |
| Severe vs. None | -0.020 | -0.072 to 0.033 | 0.054 |
| Severe vs. Mild-to moderate | -0.0004 | -0.046 to 0.046 | 0.504 |

^1^Significance results are from two way aligned rank nonparametric ANOVA with Tukey's multiple comparisons tests because of profound deviatons from a normal distribution. Confidence intervals for the means are from standard ANOVA with family-wise confidence levels. CI-confidence intervals, MR-mitral regurgitation, other abbreviations as in Table 1.

## Supplemental Table 7. Changes of ΔAVA in presence of MR adjusted for confounders

| **Comparison groups** | **Mean difference** | **95,00% CI ^1^** | **P Value^1^** |
| --- | --- | --- | --- |
| **LVEF>50% (group 1)** |  |  |  |
| Mild-to moderate vs. None | -0.014 | -0.045 to 0.018 | 0.838 |
| Severe vs. None | -0.018 | -0.042 to 0.006 | <0.0001 |
| Severe vs. Mild-to moderate | 0.029 | 0.006 to 0.053 | 0.072 |
| **LVEF 31-50% (group 2)** |  |  |  |
| Mild-to moderate vs. None | -0.030 | -0.044 to -0.016 | <0.0001 |
| Severe vs. None | 0.053 | -0.053 to 0.024 | <0.0001 |
| Severe vs. Mild-to moderate | 0.083 | 0.055 to 0.110 | <0.0001 |
| **LVEF≤30% (group 3)** |  |  |  |
| Mild-to moderate vs. None | -0.014 | -0.045 to 0.018 | 0.838 |
| Severe vs. None | 0.0054 | -0.044 to 0.057 | 1.000 |
| Severe vs. Mild-to moderate | 0.019 | -0.025 to 0.063 | 0.834 |

^1^Significance results are from ANOVA with the listed multiple comparisons tests using also adjustments by sex, age (<74, 75-79, 80-84, > 85 years), body mass index (≤25 vs. > 25 kg/m²), CI-confidence intervals, MR-mitral regurgitation, other abbreviations as in Table 1.
